# Supplementary material for: Performance of Waterborne Polyurethanes in Inhibition of Gas Hydrate Formation and Corrosion: Influence of Hydrophobic Fragments
Source: Molecules. 2020 Dec 1;25(23):5664. doi: 10.3390/molecules25235664 (PMC7730648; doi:10.3390/molecules25235664)
Supplement: Supplementary file 1 [file molecules-25-05664-s001.pdf]

molecules

MDPI

Supplementary Material

Performance of waterborne polyurethanes in inhibition of gas hydrate formation and corrosion: Influence of hydrophobic fragments

Roman S. Pavelyev[1,2], Yulia F. Zaripova[2], Vladimir V. Yarkovoi[2], Svetlana S. Vinogradova[3], Sherzod Razhabov[3], Khasan R. Khayarov[4], Sergei A. Nazarychev[1], Andrey S. Stoporev[1,2,5,6], Rais I. Mendgaziev[5], Anton P. Semenov[5], Lenar R.Valiullin[7], Mikhail A. Varfolomeev[1,2,*], Malcolm A. Kelland[8]

[1] Department of Petroleum Engineering, Kazan Federal University, Kremlevskaya Str. 18, 420008, Kazan, Russian Federation; rpavelyev@gmail.com (R.S.P.); vma.ksu@gmail.com (M.A.V.); nazarichev.sa@gmail.com (S.A.N.); stopor89@bk.ru (A.S.S.)

[2] Department of Physical Chemistry, Kazan Federal University, Kremlevskaya Str. 18, 420008, Kazan, Russian Federation; rpavelyev@gmail.com (R.S.P.); vma.ksu@gmail.com (M.A.V.); yu-ya98@yandex.ru (Y.F.Z.); waldemaryarkovoi@gmail.com (V.V.Y.); stopor89@bk.ru (A.S.S.)

[3] Department of Electrochemical Engineering, Kazan National Research Technological University, Karl Marx Str. 68, 420015 Kazan, Russian Federation; vsvet2000@mail.ru (S.S.V.); sherzodrazhabov@mail.ru (S.R.)

[4] Department of Organic Chemistry, Kazan Federal University, Kremlevskaya Str. 18, 420008, Kazan, Russian Federation; khayarov.kh@gmail.com (K.R.K.)

[5] Gubkin University, Department of Physical and Colloid Chemistry, 65, Leninsky Prospekt, Building 1, 119991, Moscow, Russian Federation; stopor89@bk.ru (A.S.S.); meda810@mail.ru (R.I.M.); semyonovanton@mail.ru (A.P.S.)

[6] Nikolaev Institute of Inorganic Chemistry SB RAS, Ac. Lavrentiev Ave. 3, 630090, Novosibirsk, Russian Federation; stopor89@bk.ru (A.S.S.)

[7] Federal center for toxicological, radiation and biological safety, Nauchnyi Gorodok 2, 420075, Kazan, Russian Federation; valiullin27@mail.ru (L.R.V.)

[8] Department of Chemistry, Bioscience and Environmental Engineering, Faculty of Science and Technology, University of Stavanger, N-4036 Stavanger, Norway; malcolm.kelland@uis.no (M.A.K.)

* Correspondence: mikhail.varfolomeev@kpfu.ru; Tel.: +7-843- 233-7977

Contents

General information S3

Methods for the preparation of compounds S5

Polyacrylamide Gel Electrophoresis S7

References S7

NMR and IR Spectra S8

General information

NMR Spectroscopy

^{1}H and ^{13}C NMR spectra were recorded on AVANCE 400 (Bruker, Germany) at operating frequency of 400 and 101.56 MHz, respectively. Chemical shifts were measured with reference to the residual protons of the solvent ($CDCl_3$). The following abbreviations are used to describe coupling: s = singlet, br s = broad singlet, br t = broad triplet, br q = broad quartet, br m = broad multiplet.

FT-IR Spectroscopy

FT-IR spectra (600–4000 cm^{-1}) were acquired using a Vertex 70 FT-IR spectrometer (Bruker, Germany) equipped with single reflection ZnSe crystal ATR accessory (MIRacle, PIKE Technologies).

Kerosene KO-25

Lighting kerosene (Technical conditions: 38.401-58-10-01, Russia) is a special liquid, which is obtained from the fractional distillation of crude oil; in the case of sulphurous oil the fractions are hydrotreated. Depending on the chemical composition and method of processing the source oil, its composition may include saturated aliphatic hydrocarbons (20-60%), naphthenic ones (20-50%), bicyclic aromatics (5-25%), unsaturated hydrocarbons (up to 2%), sulfur impurities, nitrogenous or oxygen compounds.

Main characteristics

Index name	State standard of the Russian Federation
Density:	
1. at 20 °C, kg m^{-3}, no more	795
2. at 15 °C, kg m^{-3}, no more	799

Fractional composition:<br>1. Distilled up to 200 °C, %, not less<br>2. the end of boiling of kerosene, °C, not higher	 50<br>290
Color in conventional units of KNS, no more	6
Height of non-smoking flame, mm, not less	25
Flash point in a closed crucible, °C, not lower	40
Cloud point, °C, not higher	-15
Acidity, mg KOH per 100 cm^3 of kerosene, no more	1.0
Ash content, %, no more	0.002
Mass fraction of sulfur,%, no more	0.04
test on the copper platten	withstands
Content of water-soluble acids and alkalis	absence
Content of mechanical impurities and water	absence

Methods for the preparation of compounds

Compound WPU-1 has been reported by Farhadiana A. et al. [1]

General method for the preparation WPU

The polyethylene glycol 400 (4.6 g, 11.5 mmol) and 2,2-bis(hydroxymethyl)propionic acid (2.14 g, 15.95 mmol or 01.56 g, 11.6 mmol) were mixed for 30 min at 70 °C to make a homogeneous blend. Then, isophorone diisocyanate (6.8 g, 30.6 mmol) was added and the polymerization was followed at 85 °C for 2 h; tetrahydrofuran (30 ml) was used to reduce the solution viscosity. After 2 h, N-substituted diethanolamine (2.98 mmol (1x) or 7.45 mmol (2.5x)) was dosed to the system and the reaction was continued for 5 h at 85 °C. At the end of polymerization, the temperature of the reaction mixture was decreased to 25 °C followed by triethylamine (1.2 equivalents on acid) addition to neutralize the solution. The solvent was removed in vacuo. Finally, deionized water was added to produce the WPU solution.

WPU-2

^{1}H NMR ($CDCl_3$, 400 MHz) δ 0.74-1.17 (br m, 31H), 1.21 (br t, 9H, $^{3}J_{HH}$ = 7.0, Hz), 1.51-1.75 (br m, 4H), 1.83 (br s, 2H), 2.36 (br s, 1H), 2.65-2.92 (br m, 4H), 2.98 (br q, 6H), 3.43-3.84 (br m, 33H), 4.17 (br m, 7H); ^{13}C NMR ($CDCl_3$, 101 MHz) δ 8.76, 18.40, 23.38, 25.69, 27.73, 29.81, 31.91, 35.16, 36.44, 41.84, 44.70, 45.01, 46.32, 46.99, 47.72, 54.97, 56.01, 61.67, 63.78, 63.98, 66.04, 67.25, 68.06, 69.71, 70.24, 70.59, 72.83, 77.37, 155.80, 157.00.

WPU-3

^{1}H NMR ($CDCl_3$, 400 MHz) δ 0.72-1.16 (br m, 31H), 1.20 (br t, 9H, $^{3}J_{HH}$ = 7.2, Hz), 1.52-1.74 (br m, 4H), 1.80-1.6 (br m, 2H), 2.51 (br s, 1H), 2.66-2.92 (br m, 4H), 2.97 (br q, 6H), 3.41-3.82 (br m, 30H), 4.00-4.26 (br m, 7H); ^{13}C NMR ($CDCl_3$, 101 MHz) δ 8.77, 14.16, 18.39, 20.53, 23.35, 25.67, 27.70, 29.77, 31.89, 35.14, 36.42, 41.83, 44.67, 44.99, 46.30, 46.97, 47.68, 53.32, 54.96, 61.64, 63.76, 63.96, 66.21, 67.38, 68.04, 68.37, 69.69, 70.21, 70.57, 72.82, 77.36, 155.75, 156.97.

WPU-4

^{1}H NMR ($CDCl_3$, 400 MHz) δ 0.76-1.19 (br m, 38 H), 1.23 (br t, 9H, $^3J_{HH}$ = 7.3, Hz), 1.52-1.76 (br m, 4H), 1.80-1.86 (br m, 2H), 2.66-2.92 (br m, 4H), 3.00 (br q, 6H), 3.43-3.91 (br m, 34H), 3.94-4.28 (br m, 6H); ^{13}C NMR ($CDCl_3$, 101 MHz) δ 8.74, 18.41, 18.76, 23.39, 25.69, 27.08, 27.81, 29.78, 31.92, 35.25, 36.45, 41.84, 44.70, 45.09, 46.40, 47.00, 47.69, 53.83, 54.86, 61.68, 63.82, 63.99, 66.08, 68.07, 68.37, 69.71, 70.26, 70.60, 72.80, 77.36, 156.45, 158.96.

WPU-5

^{1}H NMR ($CDCl_3$, 400 MHz) δ 0.72-1.14 (br m, 33H), 1.18 (br t, 9H, $^3J_{HH}$ = 7.2, Hz), 1.53-1.75 (br m, 4H), 1.81-1.85 (br s, 2H), 2.34 (br s, 1H), 2.58-2.75 (br m, 2H), 2.82-2,96 (br m, 8H), 3.55-3.80 (br m, 37H), 4.05-4.28 (br m, 9H); ^{13}C NMR ($CDCl_3$, 101 MHz) δ 9.23, 18.44, 23.39, 25.69, 27.80, 29.83, 31.92, 35.17, 36.45, 41.85, 42.21, 43.24, 44.70, 45.20, 46.32, 47.00, 47.69, 53.82, 54.98, 56.25, 59.12, 61.67, 62.24, 63.79, 63.99, 66.26, 67.40, 68.07, 69.72, 70.24, 70.60, 72.84, 77.37, 155.90, 157.13.

WPU-6

^{1}H NMR ($CDCl_3$, 400 MHz) δ 0.76-1.16 (br m, 49H), 1.20 (br t, 9H, $^3J_{HH}$ = 7.2, Hz), 1.24-1.44 (br m, 42H), 1.51-1.74 (br m, 4H), 1.82 (br m, 2H), 2.52 (br s, 1H), 2.66-2.91 (br m, 6H), 2.96 (br q, 6H), 3.43-3.82 (br m, 45H), 4.01-4.27 (br m, 11H); ^{13}C NMR ($CDCl_3$, 101 MHz) δ 8.83, 14.16, 18.40, 20.53, 23.36, 25.68, 27.72, 29.81, 31.90, 35.16, 36.43, 41.83, 44.69, 45.03, 46.32, 47.00, 47.67, 53.28, 54.98, 61.66, 62.92, 63.77, 63.98, 66.17, 67.36, 68.04, 69.70, 70.25, 70.59, 72.80, 77.36, 155.98, 157.27.

WPU-7

^{1}H NMR ($CDCl_3$, 400 MHz) δ δ 0.74-1.17 (br m, 40 H), 1.18 (br t, 9H, $^3J_{HH}$ = 7.1, Hz), 1.21 (br s, 1H), 1.55-1.75 (br m, 4H), 1.80-1.86 (br m, 2H), 2.72 (br s, 2H), 2.83-2.97 (br m, 9H), 3.55-3.82 (br m, 34H), 3.93-4.27 (br m, 10H); ^{13}C NMR ($CDCl_3$, 101 MHz) δ 9.11, 18.43, 23.37, 25.68, 27.12, 27.77, 29.77, 31.91, 35.16, 36.44, 41.86, 44.70, 45.14, 46.35, 47.01, 50.52, 54.97, 61.68, 63.79, 63.99, 65.77, 68.05, 69.71, 70.26, 70.60, 72.81, 77.36, 156.26, 157.43.

Polyacrylamide Gel Electrophoresis

Molecular weights and homogeneity of the polymers (1%) were estimated by running SDS-PAGE using 5% (w/v) stacking and 15% (w/v) separating gels. After electrophoresis migration, compounds bands were stained with Coomassie Brilliant Blue R-250. Molecular mass was estimated using a 3.8 kDa Standard [WPU-1, described in 1] compound and a pre-stained standard protocol (3kDa – 198 kDa) (Life technologies).

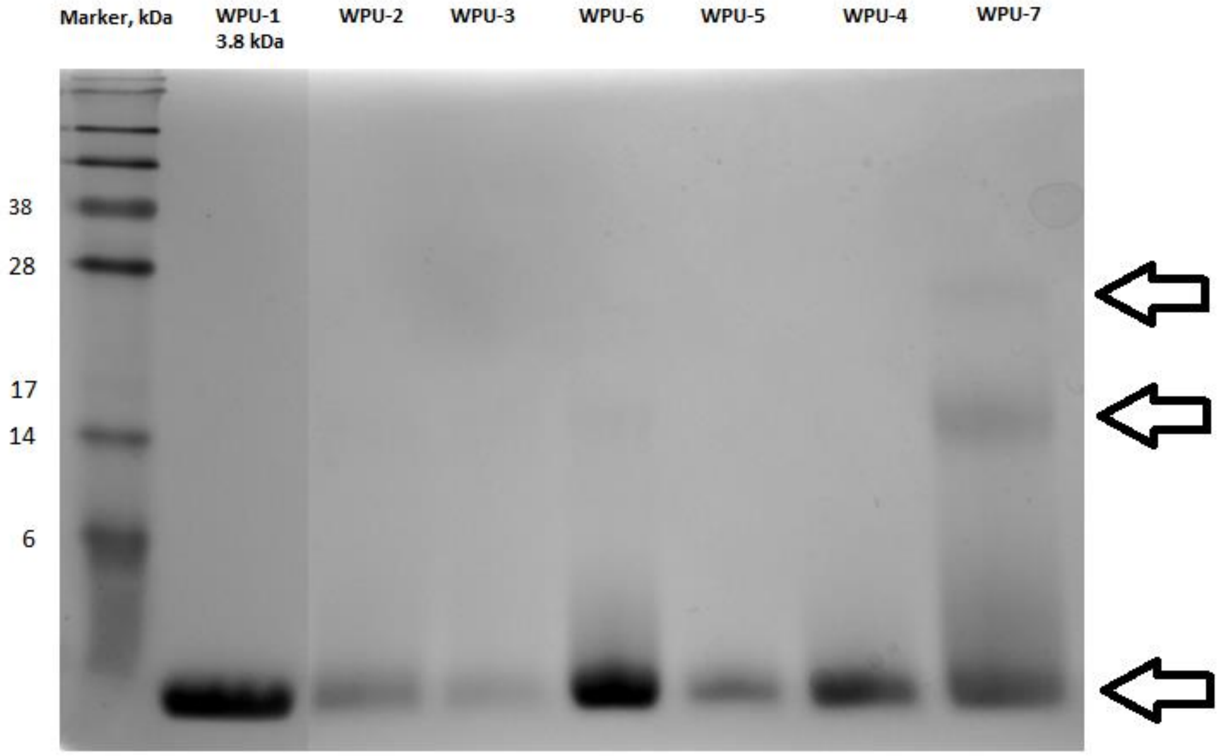

References

[1] Farhadian, A.; Kudbanov, A.; Varfolomeev, M.A.; Dalmazzone, D. *Sci. Rep.* **2019**, *9*, 9797.

NMR and IR Spectra

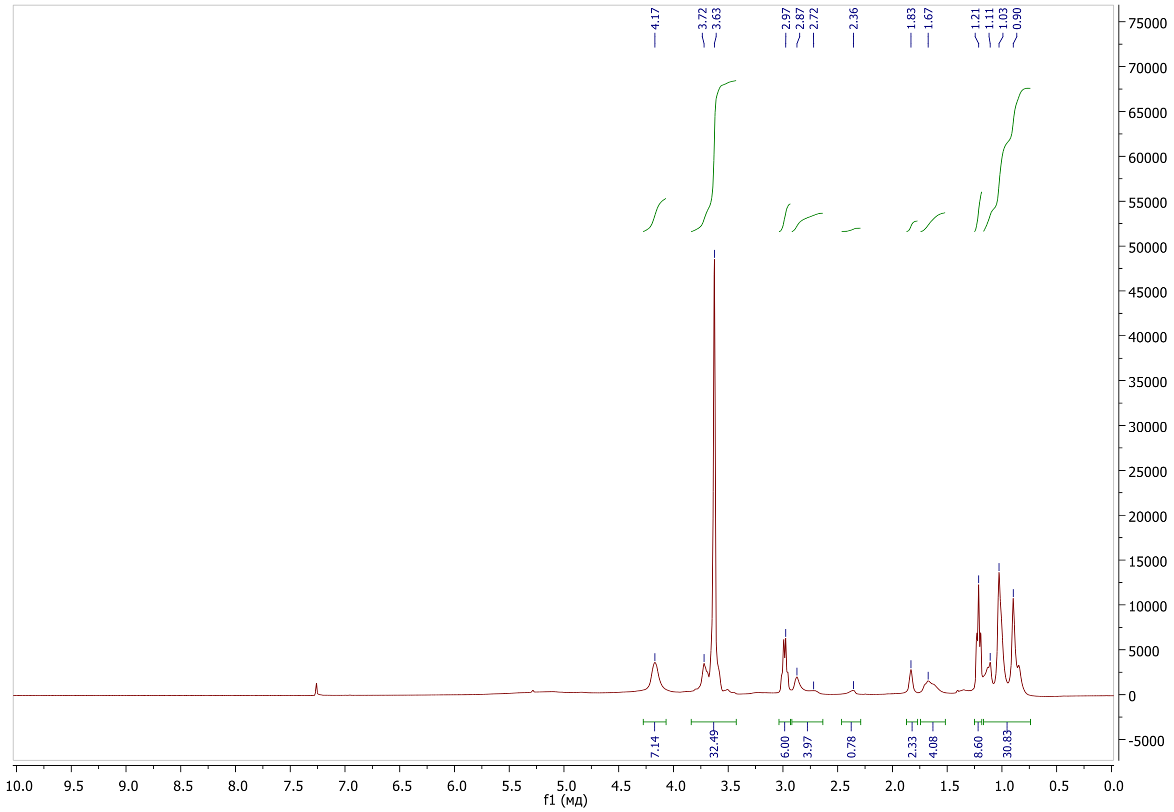

^{1}H NMR spectrum of compound **WPU-2**

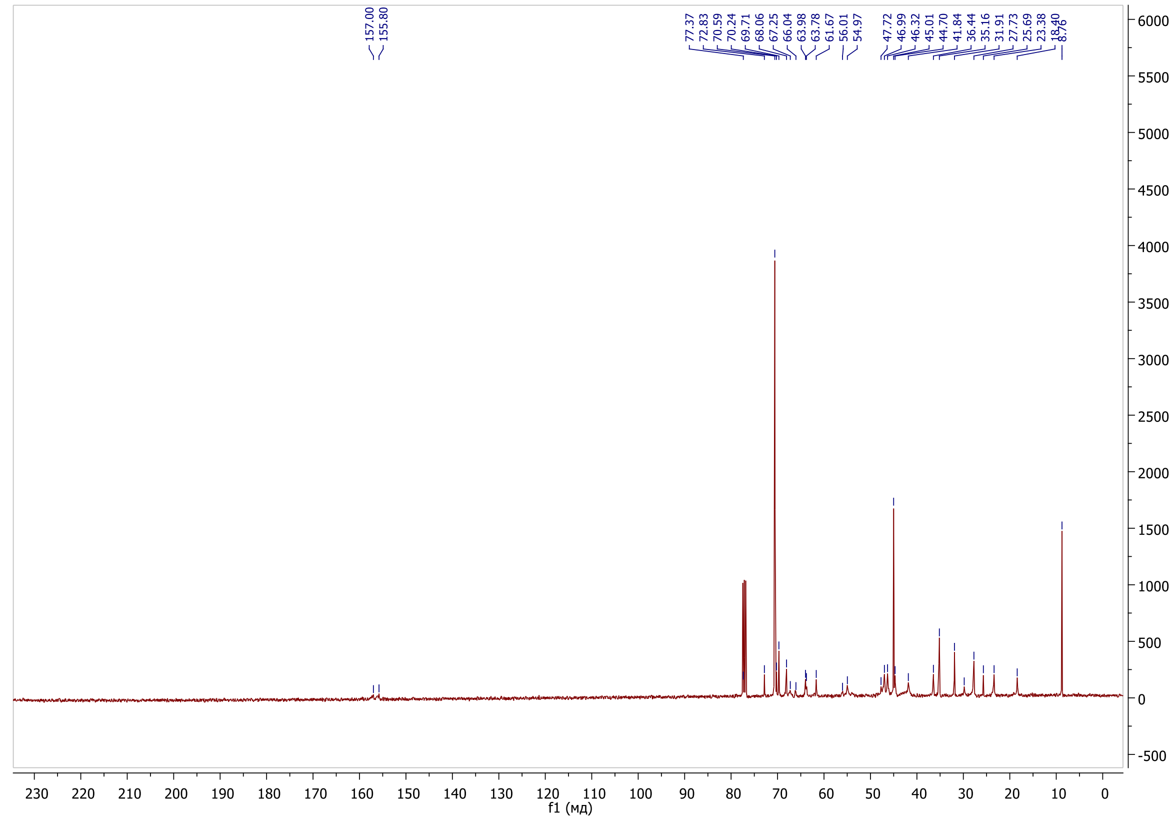

^{13}C{H} NMR spectrum of compound **WPU-2**

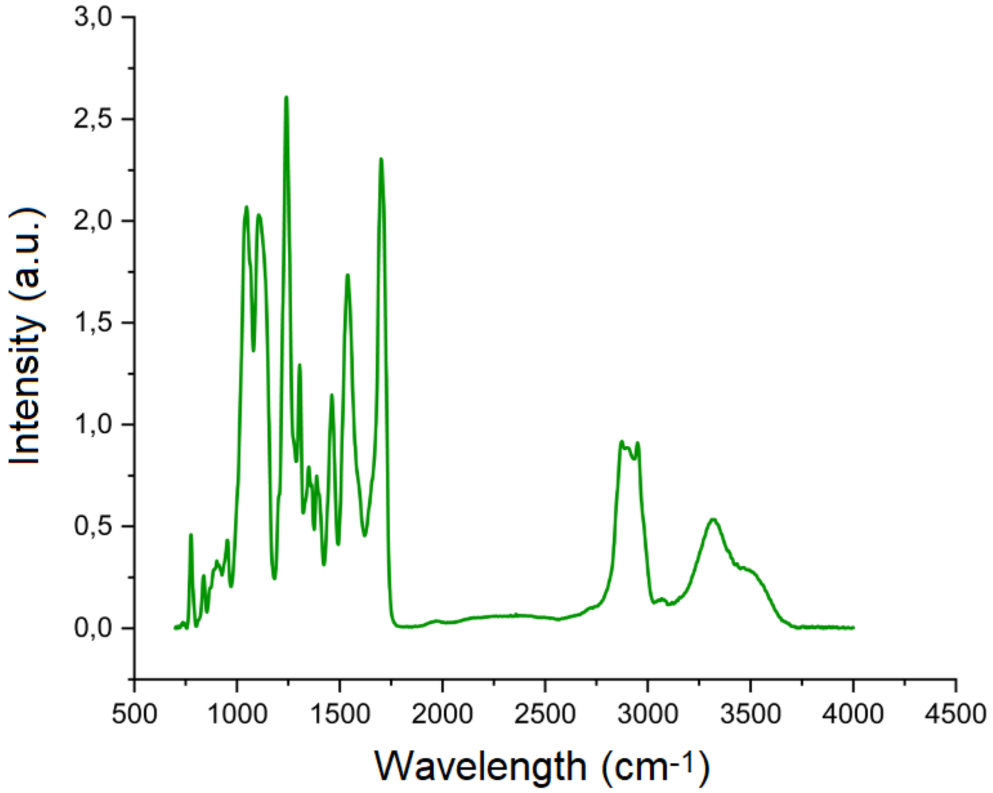

IR spectrum of compound **WPU-2**

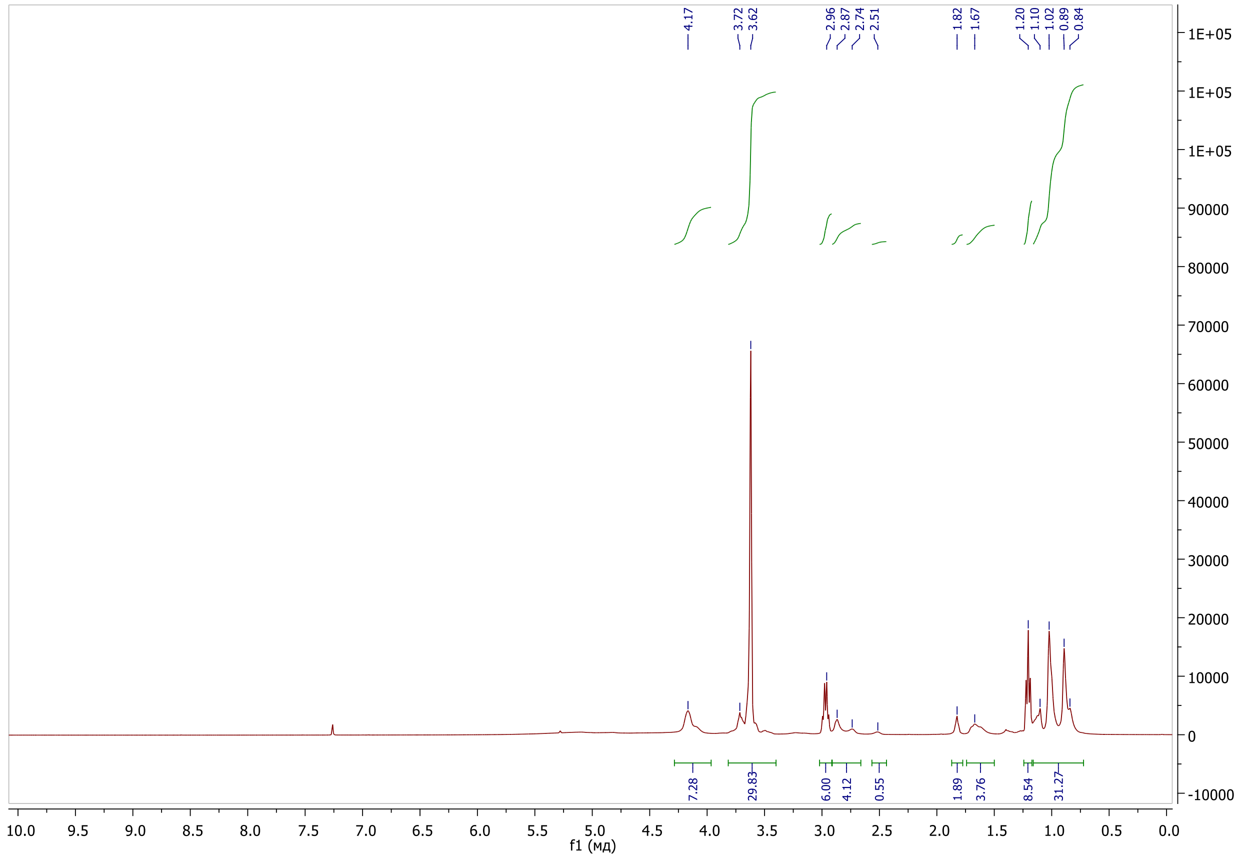

^{1}H NMR spectrum of compound **WPU-3**

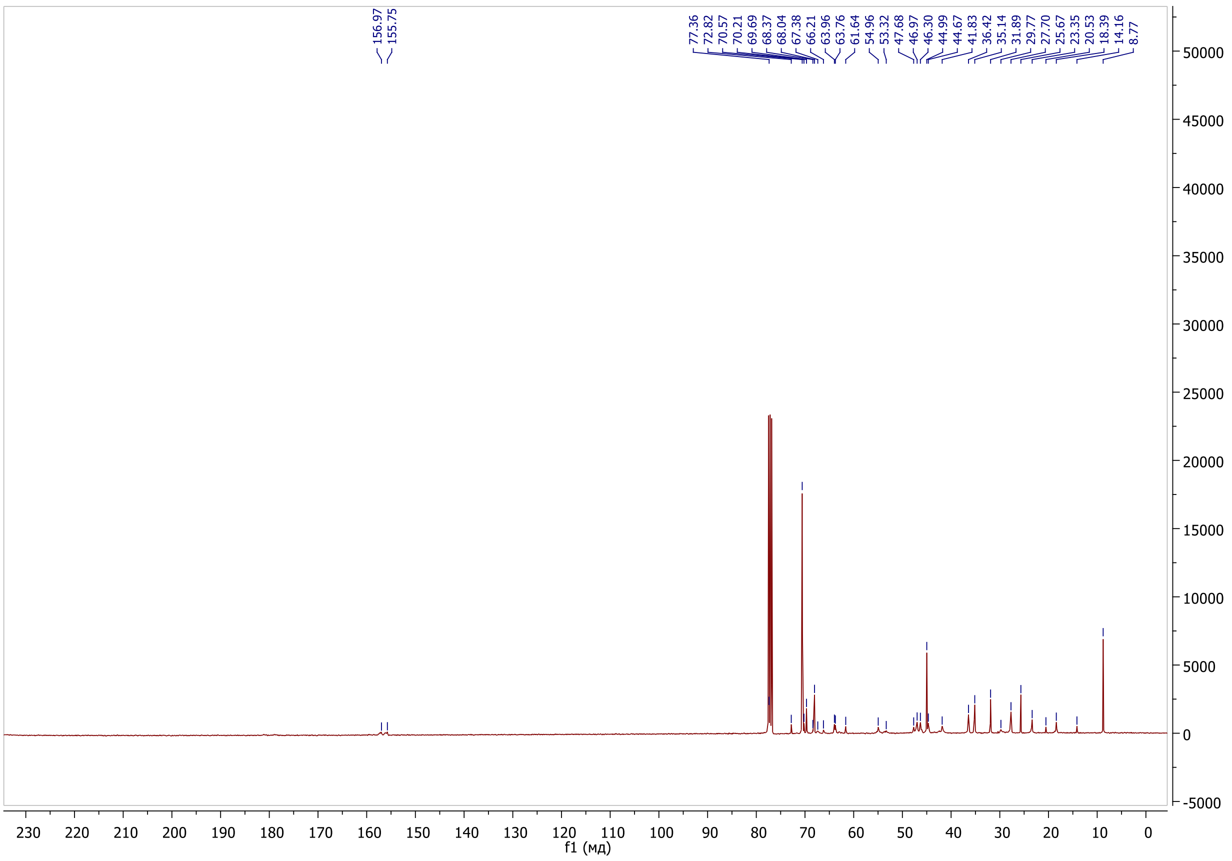

$^{13}C\{H\}$ NMR spectrum of compound **WPU-3**

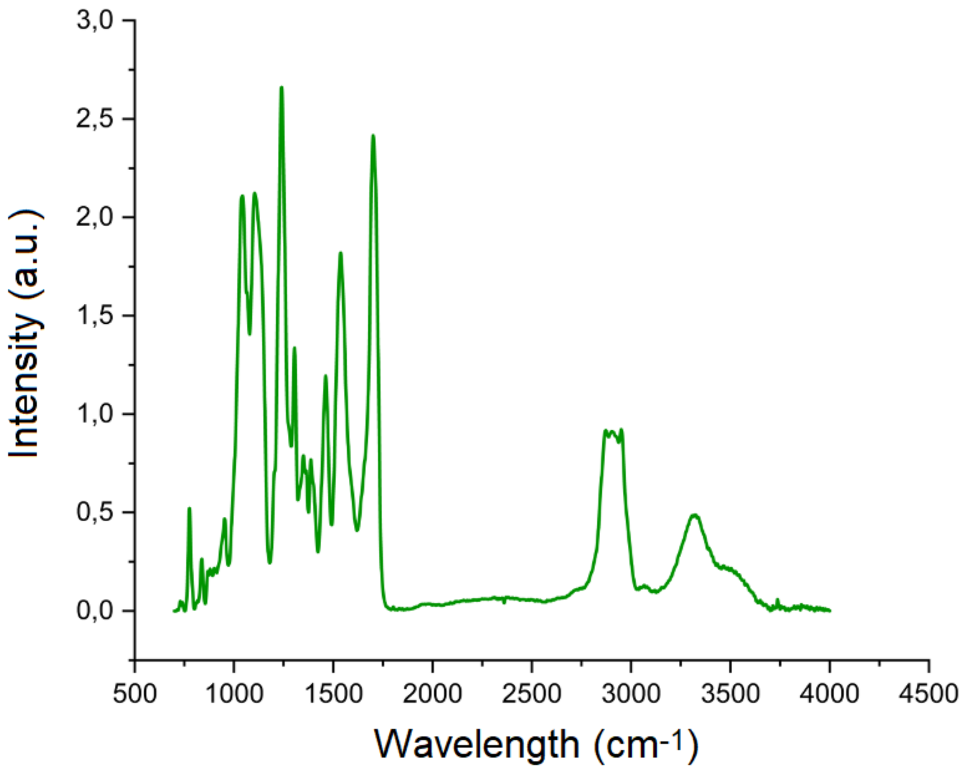

IR spectrum of compound **WPU-3**

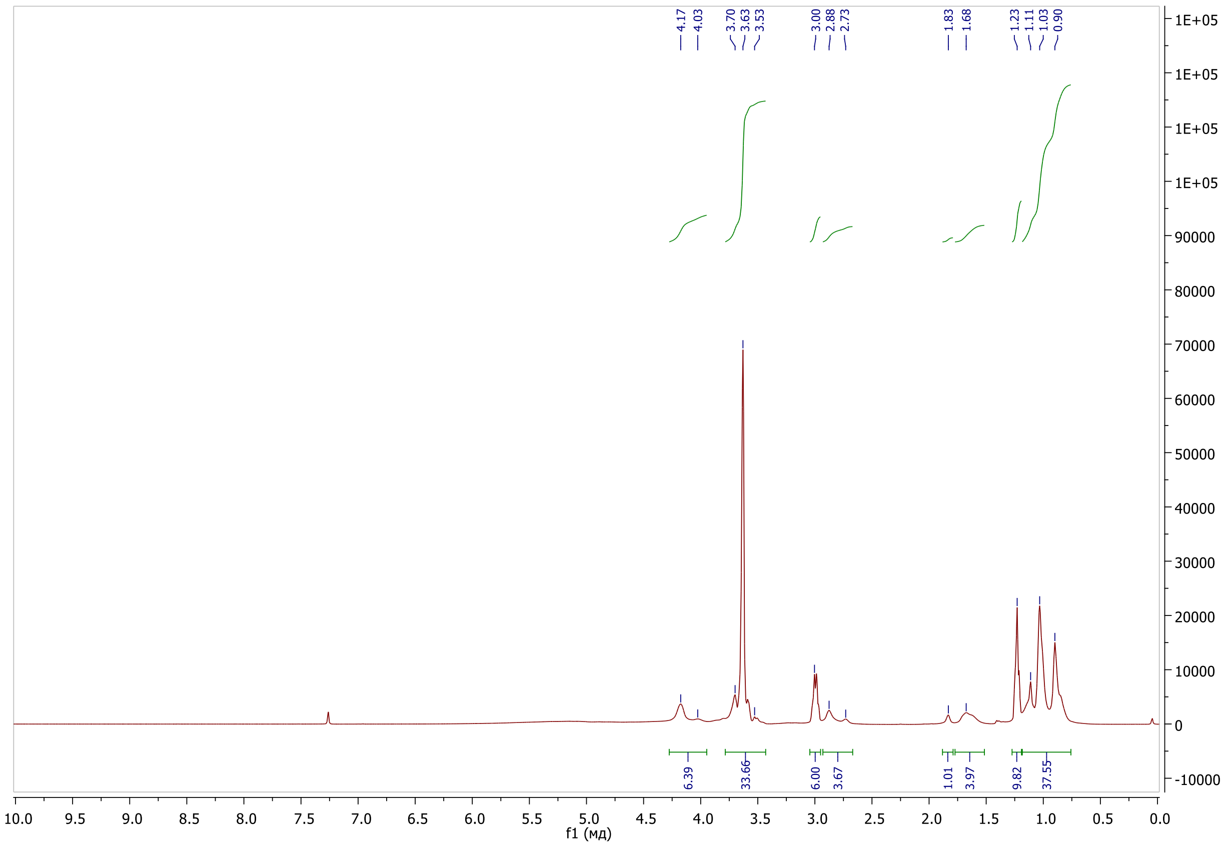

^{1}H NMR spectrum of compound **WPU-4**

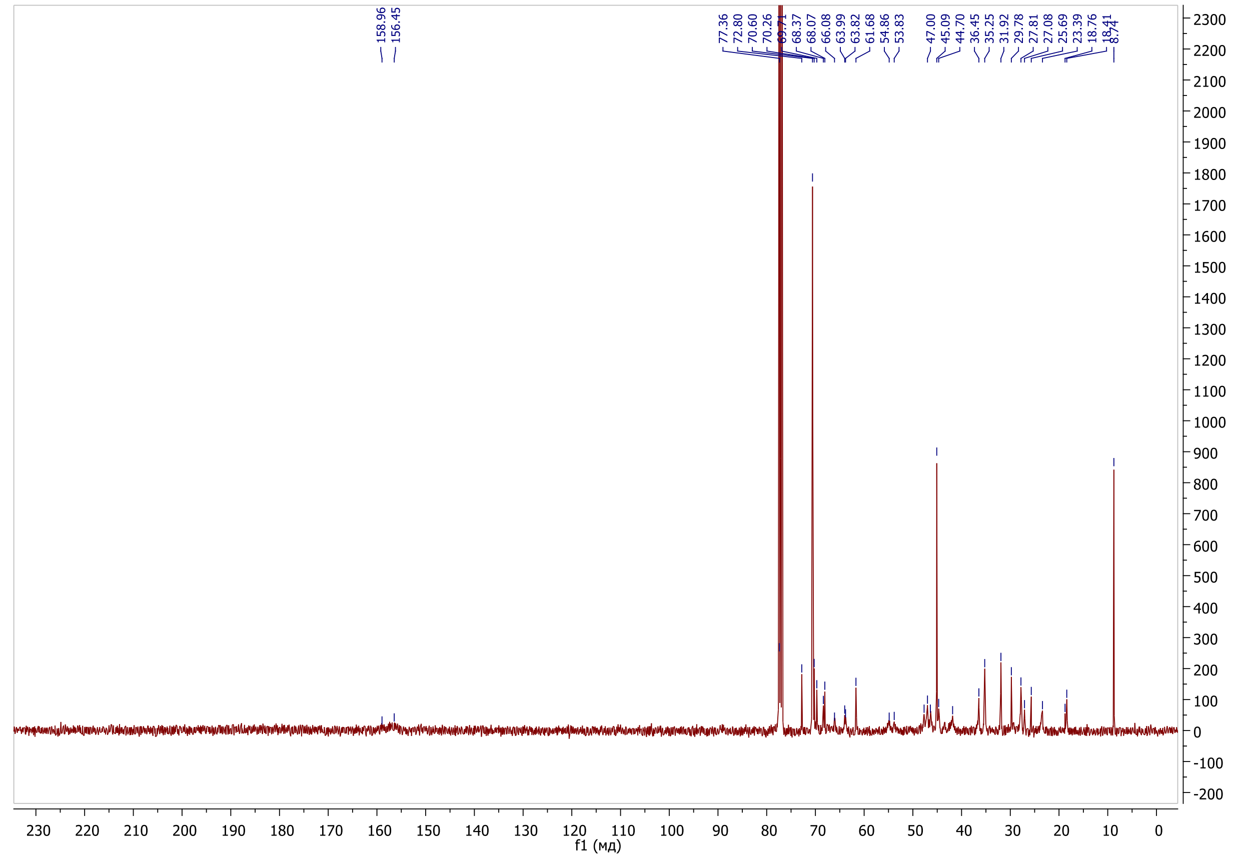

^{13}C{H} NMR spectrum of compound **WPU-4**

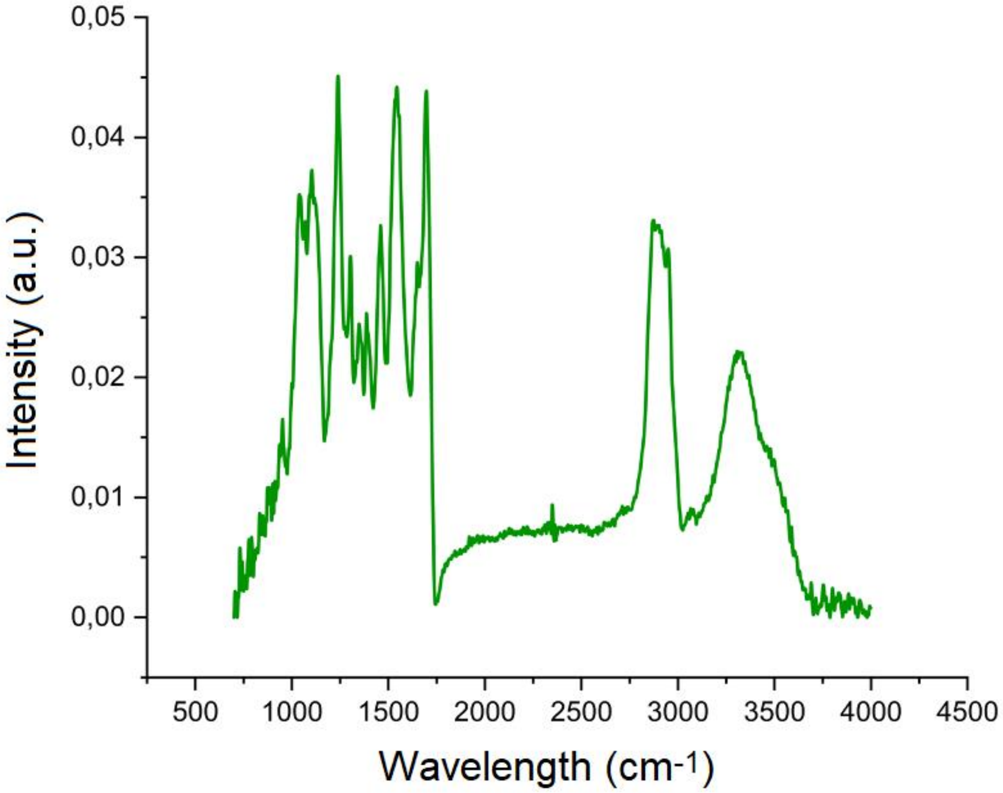

IR spectrum of compound **WPU-4**

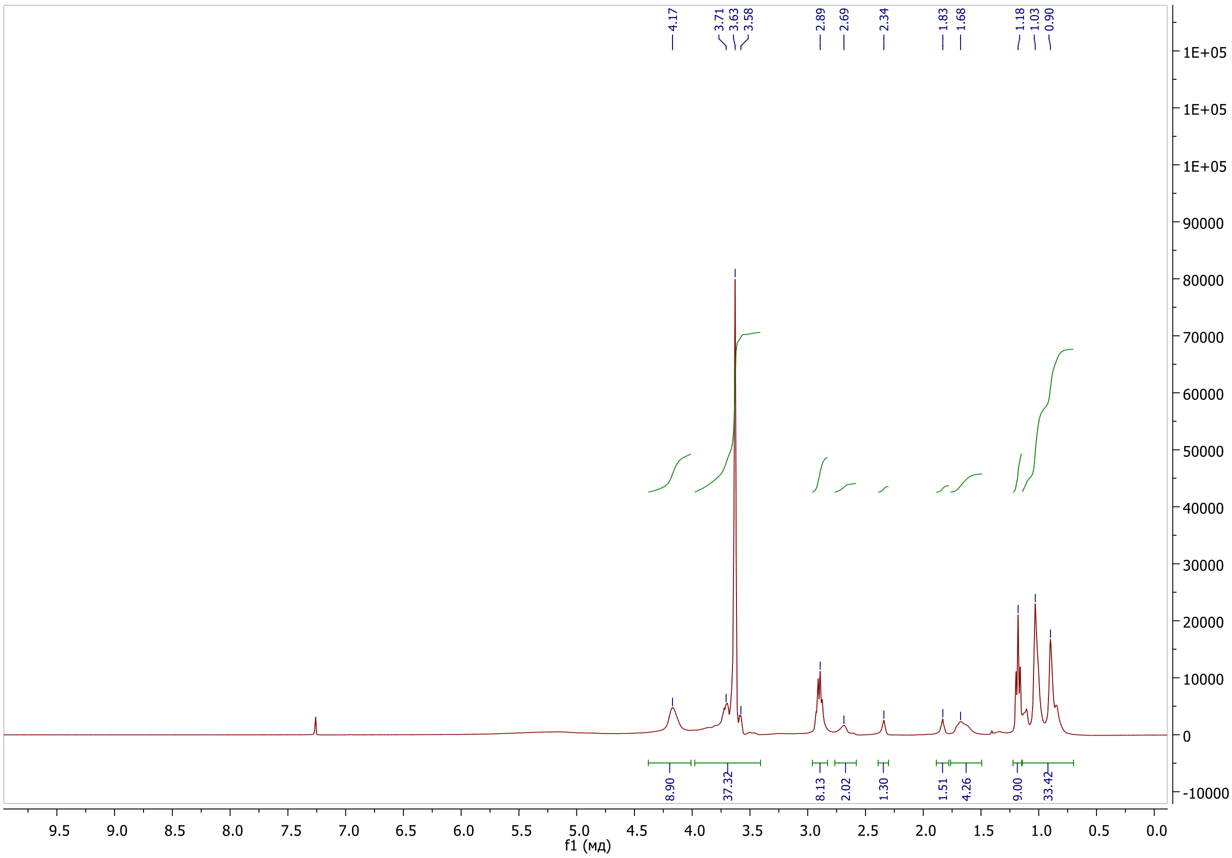

^{1}H NMR spectrum of compound **WPU-5**

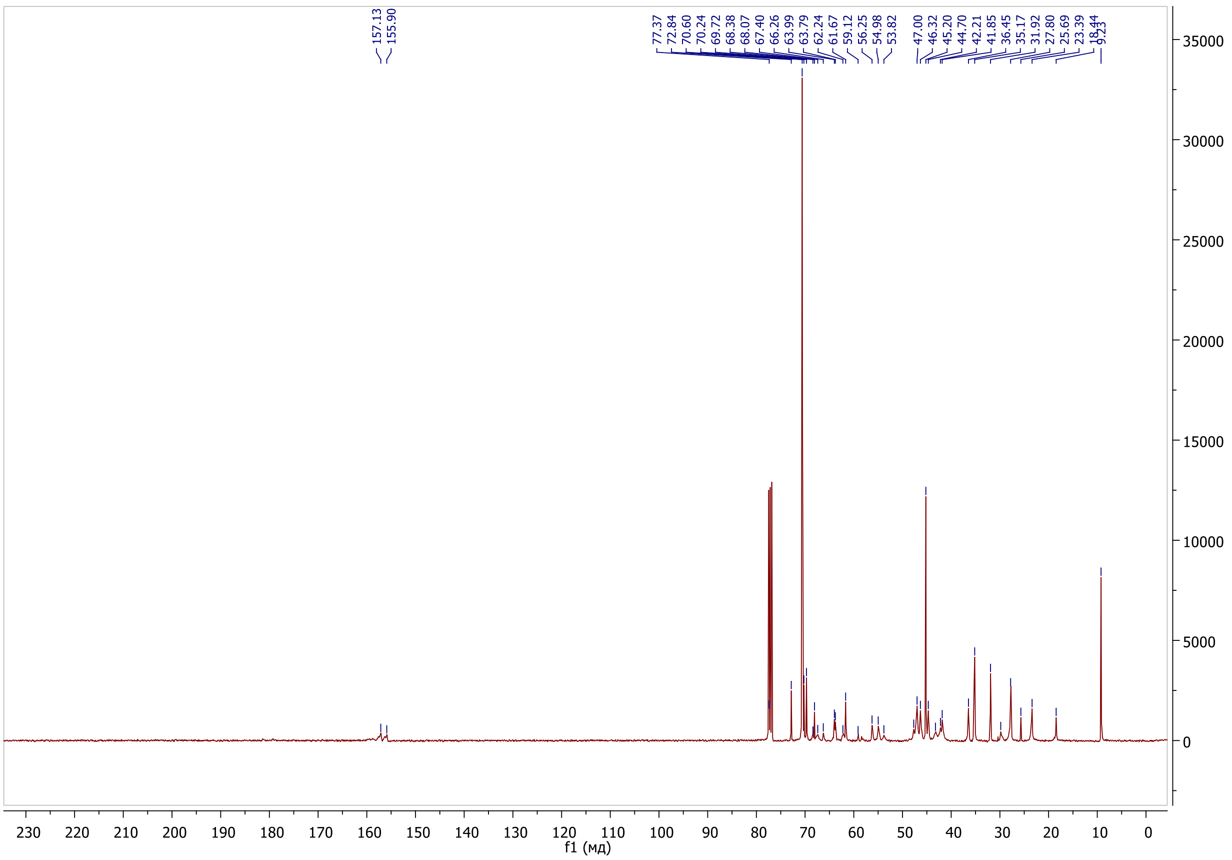

^{13}C{H} NMR spectrum of compound **WPU-5**

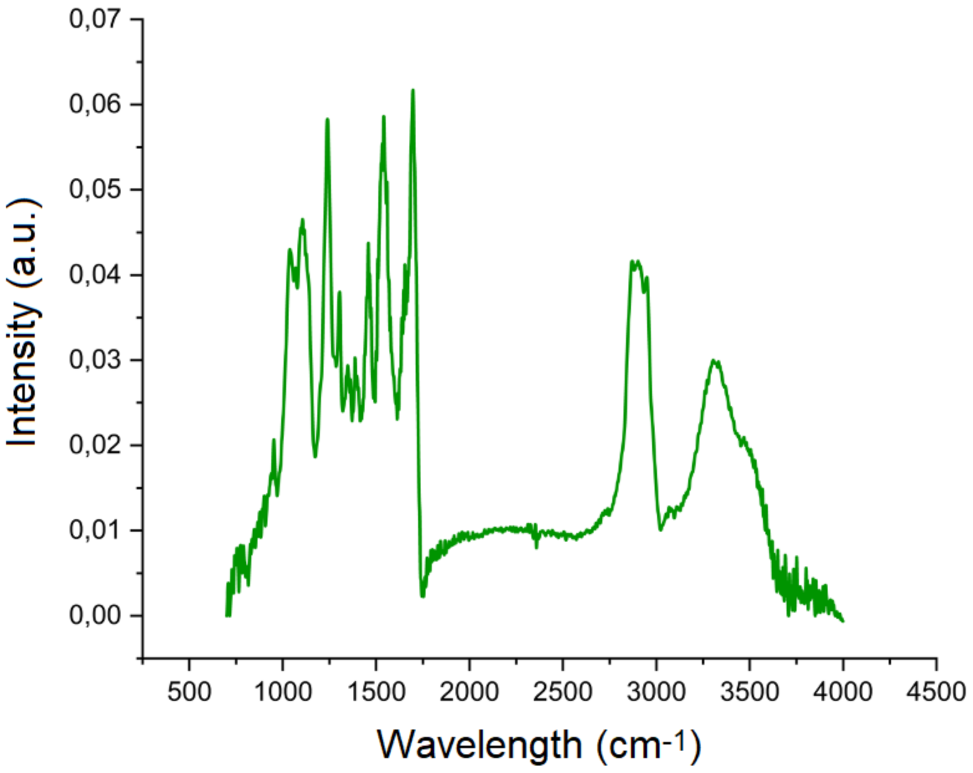

IR spectrum of compound **WPU-5**

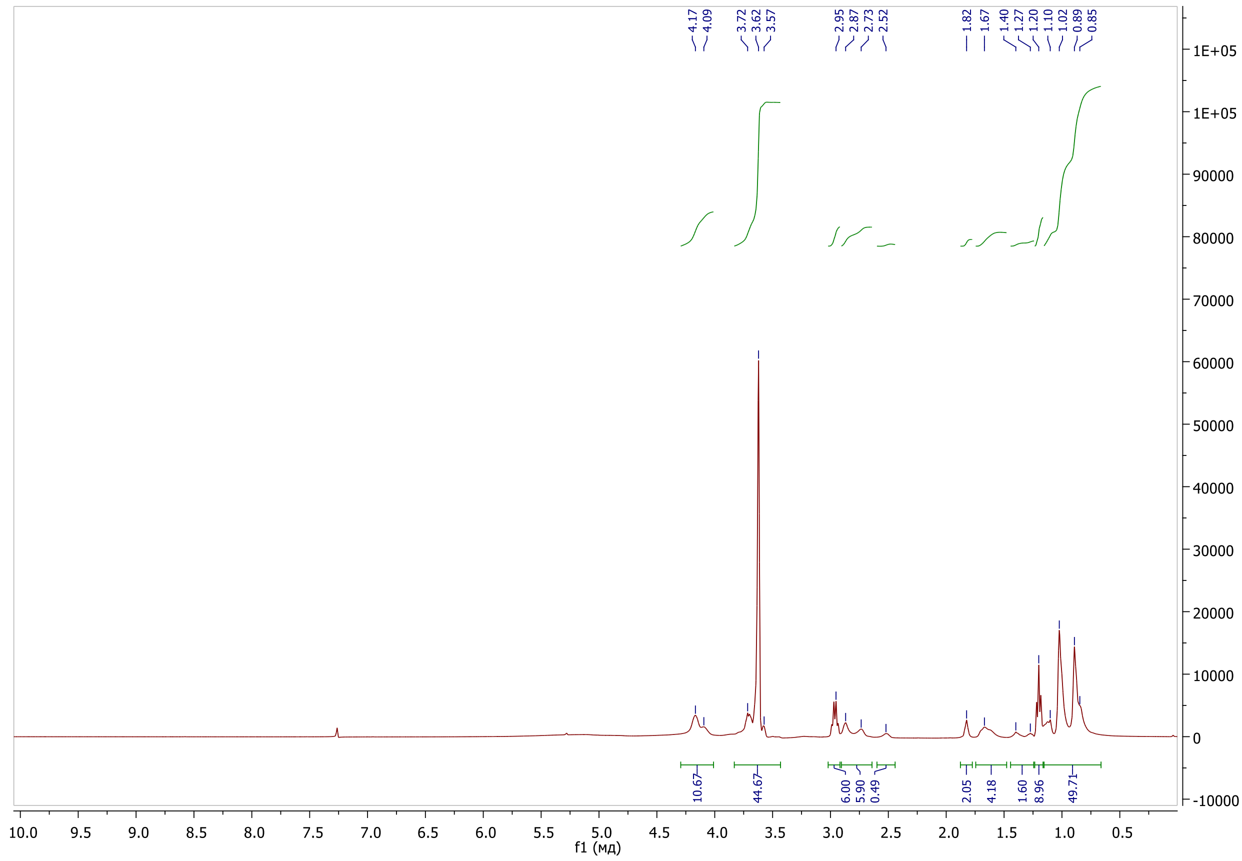

^{1}H NMR spectrum of compound **WPU-6**

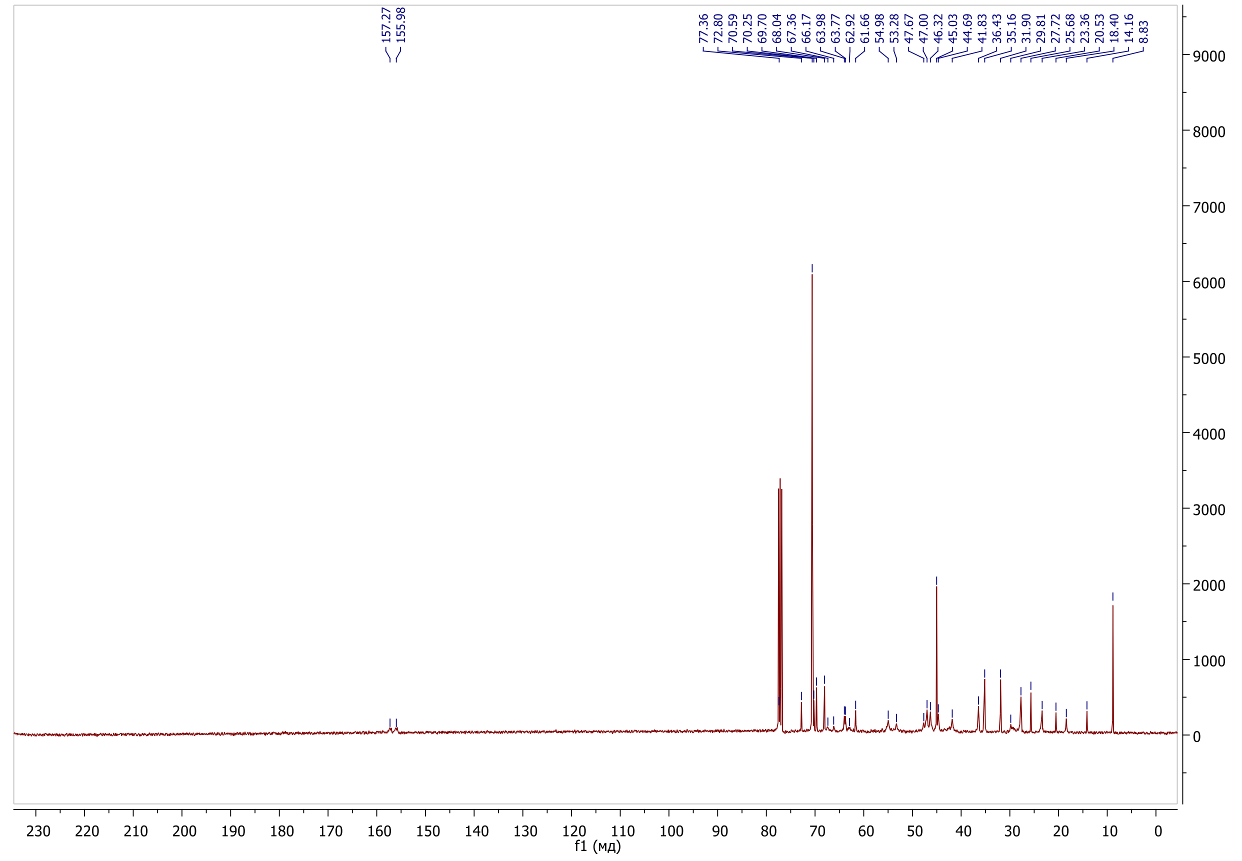

$^{13}C\{H\}$ NMR spectrum of compound **WPU-6**

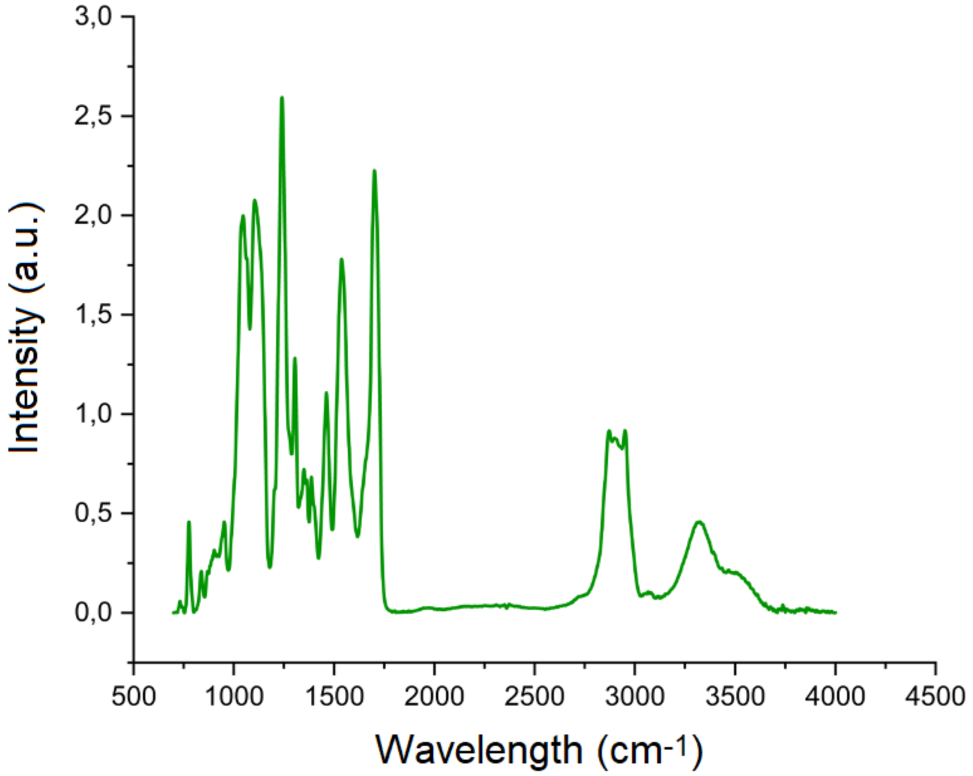

IR spectrum of compound **WPU-6**

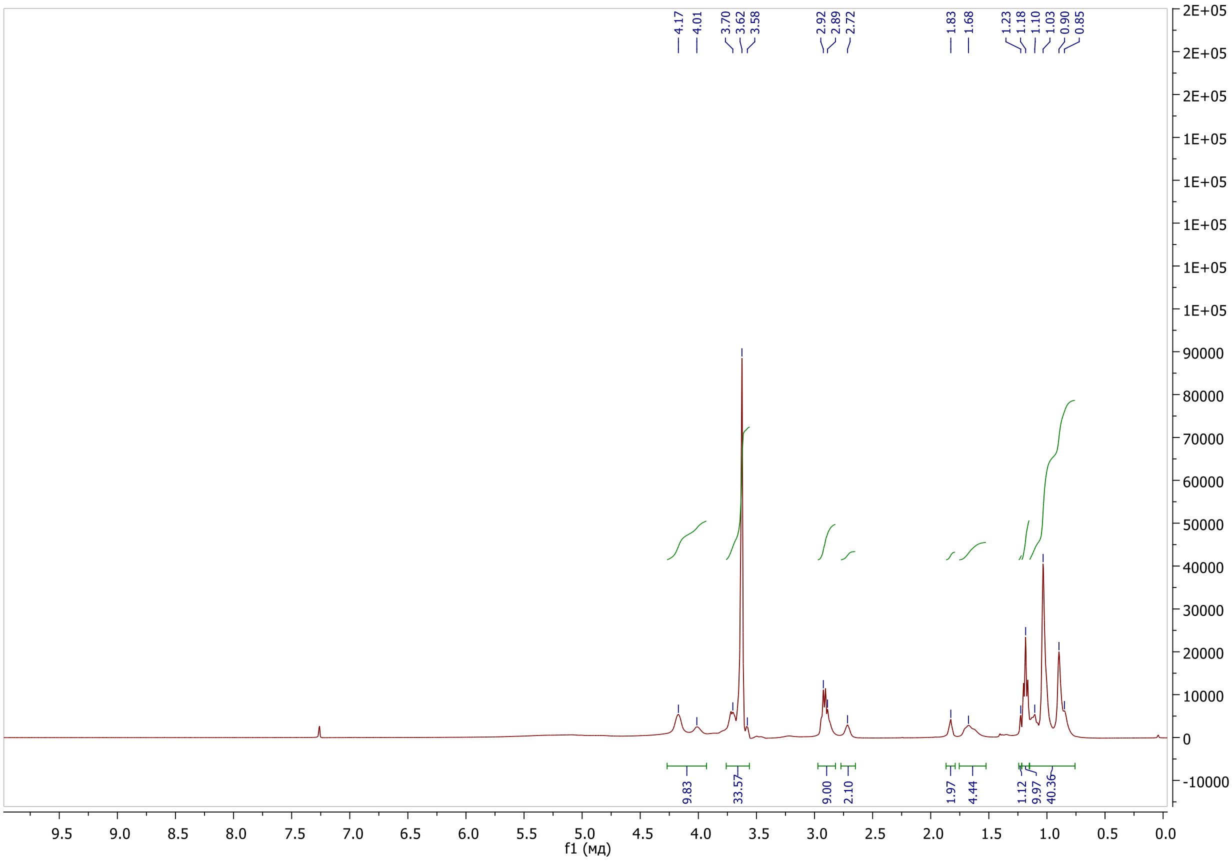

^{1}H NMR spectrum of compound **WPU-7**

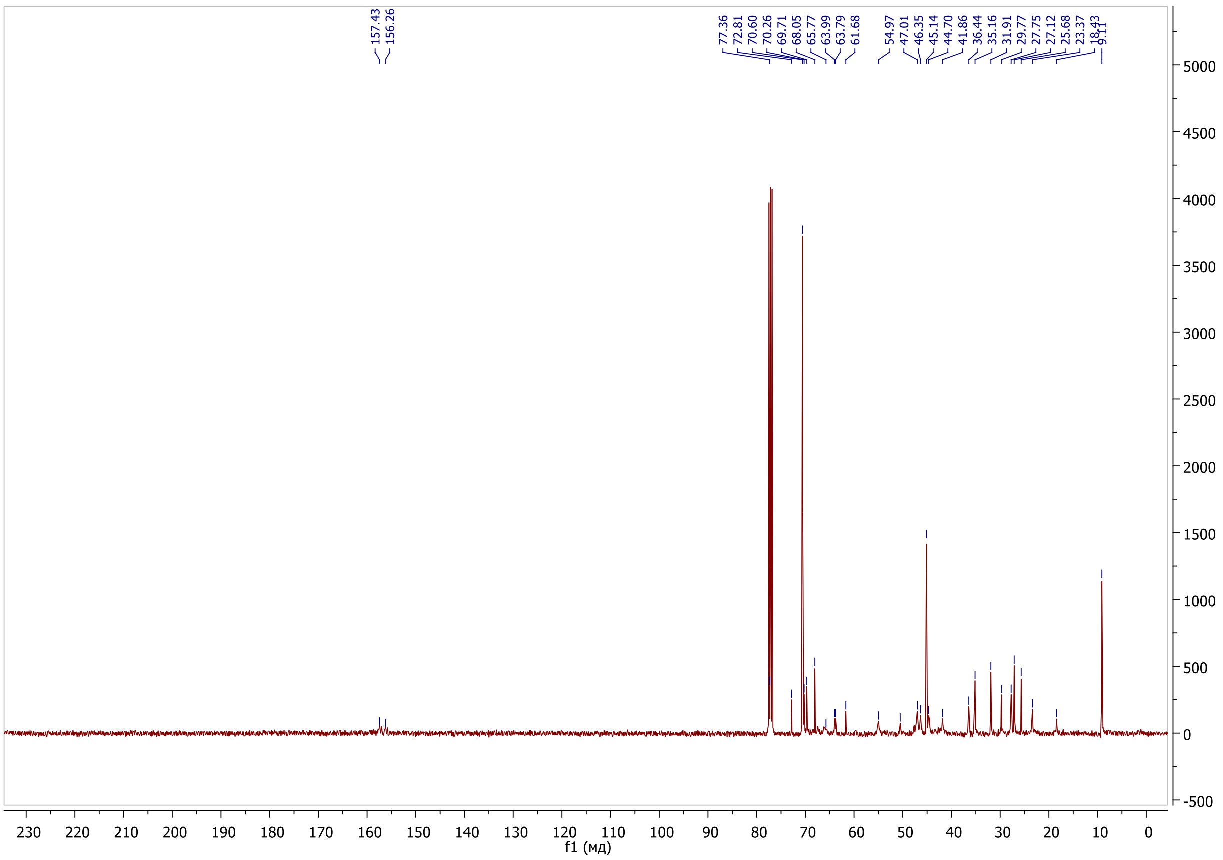

$^{13}C\{H\}$ NMR spectrum of compound **WPU-7**

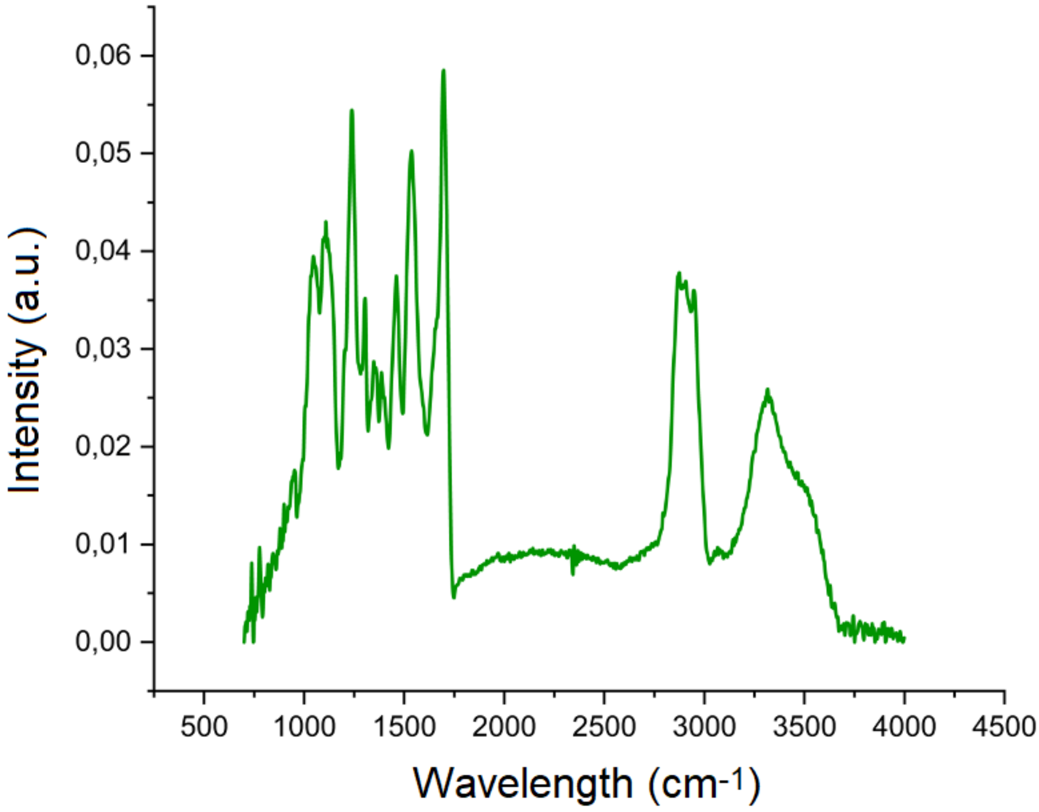

IR spectrum of compound **WPU-7**
